# The Functional Significance of Affect Recognition, Neurocognition, and Clinical Symptoms in Schizophrenia

**DOI:** 10.1371/journal.pone.0170114

**Published:** 2017-01-18

**Authors:** Charles Lung-Cheng Huang, Sigmund Hsiao

**Affiliations:** 1 Department of Psychiatry, Chi Mei Medical Hospital, Tainan, Taiwan; 2 Department of Social Work, Chia Nan University of Pharmacy and Science, Tainan, Taiwan; 3 Department of Psychology, National Chung Cheng University, Chiayi, Taiwan; University of Texas Health Science Center at San Antonio Cancer Therapy and Research Center at Houston, UNITED STATES

## Abstract

**Objectives:**

The complex relationship and exact extent of the contribution of plausible indictors to social functional outcome in schizophrenia remain unclear. The present study aimed to explore the functional significance of clinical symptoms, neurocognition, and affect recognition simultaneously in schizophrenia.

**Methods:**

The clinical symptoms, basic neurocognition, facial emotion recognition, and social functioning of 154 subjects, including 74 with schizophrenia and 80 nonclinical comparisons, were assessed.

**Results:**

We observed that various subdomains of social functioning were extensively related to general intelligence, basic neurocognition, facial emotion recognition, and clinical symptoms, with different association patterns. Multivariate regression analyses revealed that years of education, age, sustained attention, working memory, and facial emotion recognition were significantly associated with global social functioning in schizophrenia.

**Conclusion:**

Our findings suggest that affect recognition combined with nonsocial neurocognition demonstrated a crucial role in predicting global social function in schizophrenia.

## Introduction

Deficits in various domains of social function are marked in patients with schizophrenia [1–2]. Therefore, many recent studies have attempted to determine indicators that can predict functional outcomes in this chronic and devastating disorder. Studies have demonstrated that impairments in social functioning are related to clinical psychopathology, especially negative symptoms, in individuals with schizophrenia [3]. In addition, neurocognition such as attention, memory, and executive function, have been frequently reported to associate with such impairments experienced by these patients [4], although interaction with clinical symptoms have also been found [5]. A growing body of literature has explored the functional significance of affect recognition [6], a domain of social cognition, and revealed that affect recognition performance may not only interact with clinical symptoms and basic neurocognition [7], but also exceed the value of nonsocial neurocognition in explaining functional outcome in schizophrenia [8].

Notwithstanding the plausible causal links and complex relationships among clinical symptoms, neurocognition, and affect recognition, the exact extent of the contribution of these factors to social functional outcome remains unclear. In brain basis, the independence/interdependence between emotional perception and neurocognition has long been be debated because interconnection between the emotion-processing limbic area and execution-related medial frontal area has been reported. In addition, even though some researchers argued that social function impairment in patients with schizophrenia is owing to their failure to realize the meaning of perceived emotional cues rather than disabilities in affect recognition [9], there were postulates that difficulties in an earlier phase of emotion perception may have influence on the down-stream process of judging emotional implications [10], thus leading to dysfunction in social problem solving and community functioning.

To untangle these complex interrelationships, it is necessary to take all these factors into consideration simultaneously when investigating their functional significance. Models concerning the interrelation among clinical symptoms, neurocognition, and affect recognition can offer a notional frame for comprehending the underlying mechanism and brain basis of social function impairment in schizophrenia [11–12]. Supposing the nature of these underlying factors and brain basis can be illuminated, management including prevention, treatment, and rehabilitation can be developed and applied to ameliorate symptoms and improve patient outcomes. Although previous studies have reported separate relationships between these variables in people with schizophrenia, few of them have examined the functional significance of these interactive factors altogether. Furthermore, many studies found a relationship between functional outcome and other factors, but did not specify which domain of social function was related. To address the gaps in the existing literature, the goal of this study was to illustrate the integrated picture of social functional outcome in patients with schizophrenia by examining the interrelationships among clinical symptoms, neurocognition, affect recognition, and social function. We aimed to determine the functional significance of clinical symptoms, neurocognition, and affect recognition simultaneously in schizophrenia.

## Materials and Methods

### Subjects and design

This study was implemented in a general hospital located in central Taiwan. One hundred fifty-four subjects, including 74 with schizophrenia (44 paranoid, 18 disorganized, and 12 other subtypes) and 80 nonclinical comparisons, participated in the study.

Schizophrenia patients were recruited from the inpatient ward, day-care units, and outpatient clinic. They had to meet the criteria for schizophrenia in the DSM-IV-TR [13], as assessed by the Structured Clinical Interview for DSM-IV (SCID-P) by psychiatrists [14]. At the time of the study, all of the patients were clinically stable under medication treatment. Nonclinical comparison subjects were enrolled from the community. They had no psychiatric disorders as assessed by SCID-P criteria and had no familial history of a psychotic disorder.

All participants were between 18 and 50 years old. They were excluded from the study if they met DSM-IV criteria for mental retardation, pervasive developmental disorder, or substance dependence, had a documented ophthalmic or neurological condition, or had ever received electroconvulsive therapy.

### Ethics statement

The study was approved by the Research Ethics Committee of National Taiwan University Hospital (approval No. 200909037R). All subjects participated after completing written informed consent, and all methods were performed in accordance with the Helsinki Declaration of 1975, as revised in 2008.

### Measures

#### Clinical assessment

The Chinese version of the Positive and Negative Syndrome Scale (PANSS) was used to measure severity of clinical symptoms in patients with schizophrenia [15–16]. The PANSS is a 30-item semistructured interview that provides a composite estimate for global symptoms severity from three subscales which rate positive symptoms (PANSS-Positive), negative symptoms (PANSS-Negative), and general psychopathology (PANSS-General). All items are scored between 1 (not present) and 7; a higher score indicates more severe symptoms. The Chinese version has been shown to be reliable and valid in practice [16]. In order to control the possible influence of depressive symptoms on the performance of emotion recognition [17], depressive symptoms among participants were assessed using the Calgary Depression Scale for Schizophrenia and Beck Depression Inventory for nonclinical comparisons [18–19].

#### Neuropsychological assessment

The Chinese version of Raven's Standard Progressive Matrices (SPM) was used to assess participants’ general intelligence [20–21]. The nonverbal aspect of the test minimizes the impact of cultural or language bias. It is a 60-item test of observation skills and clear-thinking ability used in measuring abstract reasoning and is regarded as a non-verbal estimate of fluid intelligence. The test comprises five sets (A to E) of 12 items each, with items within a set becoming increasingly difficult, requiring ever greater cognitive capacity to encode and analyze information. A higher score indicates better general intelligence development.

Sustained attention was assessed with the Chinese version of computerized Conners' Continuous Performance Test, version 2.0 (CPT-II) [22–23]. Briefly, clients are told to click the space bar when they are presented with any letter except the letter "X". The person must refrain from clicking if they see the letter "X" presented. The sensitivity index *d′* was derived from the hit rate (probability of response to target trials) and false-alarm rate (probability of response to nontarget trials), which measures an individual's ability to discriminate target stimuli from nontarget stimuli.

The Chinese version of the Digit Span Test (DS) was used to measure working memory’s number storage capacity [24]. It is also a subset of the Wechsler Memory Scale [25]. Participants are presented with a series of digits and must immediately repeat them back. If they do this successfully, they are given a longer list. The length of the longest list a person can remember is that person's digit span. In the forward digit-span task, the participant is asked to enter the digits in the given order and in the backward digit-span task the participant needs to reverse the order of the numbers.

Executive functioning was assessed using the Chinese version of computerized Wisconsin Card Sorting Test (WCST), which assesses the participants’ ability to shift cognitive sets in response to performance feedback [26–27]. Briefly, four stimulus cards incorporate three stimulus parameters (color, form, and number). Respondents are required to sort numbered response cards according to different principles and to alter their approach during test administration. Scoring includes total errors, total correct, and categories completed.

#### Facial emotion recognition task

The computerized Chinese Facial Emotion Recognition Database (CFERD) was used to assess the accuracy of recognition of the 6 basic human emotions, i.e., happiness, sadness, disgust, fear, anger, and surprise [28]. Our previous work has reported and described the CFERD’s development and validation in details [28]. Briefly, the measure consists of 130 static color photographic pictures of facial emotional expressions, which are morphed between neutral and extreme emotional expressions in each of the six emotional categories in 10% steps. Each photo is shown in random order on a 15-in laptop computer screen for 3000 ms, followed by a choice list. The participants are asked to match each face with the emotion category among the 6 basic emotions by pressing the corresponding key on the keyboard. The sensitivity index (discrimination index, *d'*) is used as the measure of performance for accurate recognition of each emotional expression. A higher *d'* indicates that the signal is more readily detected, i.e., there is better ability to differentiate the target emotion from other emotions.

#### Social function assessment

Subjects’ social functioning was assessed with an adapted Chinese version of the Social Functioning Scale (SFS), a self-rated or caregiver-rated measure commonly used to measure social function in patients with schizophrenia [29–30]. The SFS provides a composite estimate of global social functioning from 7 separate social functional domains, including (1) social engagement (time spent alone, initiation of conversations, and social avoidance), (2) interpersonal behavior (number of friends, whether the patient has a partner, and quality of communication), (3) prosocial activities (engagement in a range of common social activities), (4) recreation (engagement in a range of common hobbies, interests etc.), (5) independence-competence (ability to perform skills necessary for independent living), (6) independence performance (performance of skills necessary for independent living) and (7) employment (engagement in structured employment or a structured program of daily activity). Higher scores indicate better social functioning. The scale and subscales have acceptable internal consistency and inter-rater (between parents) reliability, the former ranging from 0.69 to 0.87, the latter from 0.62 to 0.99 [29]. The scale also has good discriminant validity; it can differentiate patients from non-clinical subjects in a community population. The Chinese version contains 36 items and has been shown to be reliable and valid in practice [29].

### Data analyses

Group comparisons between patients with schizophrenia and healthy controls were performed using the chi-square test for categorical variables and two-tailed t-tests for continuous variables. Associations between clinical symptoms, basic neurocognitive measures, facial emotion recognition, and social function were explored by nonparametric correlational analyses (Spearman *ρ*). Multivariate regression analysis that employed the stepwise method was further used to determine factors that potentially contribute to global social function (total SFS score). The independent variables included in the multivariate regression analysis were as follows: age, years of education, PANSS total score, SPM score, CPT *d'*, WCST category completed, DS, and facial emotion recognition sensitivity index (FER *d'*). All analyses were two-tailed with an alpha level of *p* <0.05 All statistical comparisons were performed using the SPSS for Windows package, Release 15 (SPSS; Chicago, IL, USA).

## Results

### Study participant characteristics

Subjects with schizophrenia and nonclinical comparisons were comparable in gender and age although they differed in educational level (Table 1). Patients with schizophrenia had poorer performances in facial emotion recognition and all neurocognitive measures. The mean social function total and domain scores were lower in the schizophrenia group than the controls (Table 1). The subgroup analysis showed that patients with non-paranoid schizophrenia have lower social functioning than paranoid schizophrenia and health controls (*p* < 0.05).

**Table 1:** Demographic data, clinical characteristics, facial emotion recognition, neurocognitive performances, and social function in schizophrenia and nonclinical comparisons.

	Schizophrenia (N = 74)	Comparisons (N = 80)	Analysis	
	n (%)	n (%)	d.f.	Χ^2^
**Male gender**	31 (42)	40 (50)	1	1.02
	mean ± SD	mean ± SD	d.f.	*t*
**Age (years)**	33.22 ± 8.23	30.81 ± 6.83	152	-1.96
**Educational level (years)**	12.02 ± 1.78	15.84 ± 2.01	152	12.41**
**Age of onset (years)**	22.64 ± 6.56	N/A	N/A	N/A
**Duration of illness (years)**	10.65 ± 7.70	N/A	N/A	N/A
**Number of hospitalizations**	4.93 ± 4.70	N/A	N/A	N/A
**Chlorpromazine equivalents (mg)**	445.12 ± 324.37	N/A	N/A	N/A
**PANSS**				
Positive symptoms	18.96 ± 4.20	N/A	N/A	N/A
Negative symptoms	19.81 ± 3.62	N/A	N/A	N/A
General psychopathology	34.99 ± 5.63	N/A	N/A	N/A
Total score	73.76 ± 10.28	N/A	N/A	N/A
**CDSS score**	1.08 ± 1.38	N/A	N/A	N/A
**BDI score**	N/A	1.28 ± 1.03	N/A	N/A
**FER *d’***	0.73 ± 0.26	0.93 ± 0.23	152	5.16**
**Neurocognitive measures**				
SPM	46.90 ± 8.07	57.67 ± 2.05	152	6.70**
CPT *d’*	0.65 ± 0.34	0.78 ± 0.44	152	2.06*
WCST category	5.16 ± 3.24	7.49 ± 2.53	152	4.94**
Digit span	19.80 ± 4.49	25.91 ± 3.81	152	8.61**
**Social Functioning Scale**				
Social engagement/withdrawal	3.81 ± 1.19	4.96 ± 1.12	152	6.19**
Interpersonal communication	6.61 ± 2.07	8.29 ± 1.40	152	5.85**
Independence/competence	12.39 ± 2.69	14.75 ± 0.65	152	7.35**
Independence/performance	8.65 ± 3.32	13.75 ± 1.61	152	11.99**
Recreation	14.77 ± 6.02	19.90 ± 2.95	152	6.63**
Prosocial act	6.84 ± 3.50	11.51 ± 3.39	152	8.42**
Employment/occupation	4.27 ± 2.06	5.43 ± 0.78	152	4.54**
Total score	57.34 ± 14.45	78.59 ± 7.95	152	11.18**

PANSS = Positive and Negative Syndrome Scale; CDSS = Calgary Depression Scale for Schizophrenia; BDI = Beck Depression Inventory; FER = Facial emotion recognition; SPM = Raven’s Standard Progressive Matrices; CPT = Continuous Performance Test; WCST = Wisconsin Card Sorting Test

*p < 0.05;

**p < 0.01

### Correlational analyses of Facial Emotion Recognition (FER) *d'*

The FER *d'* was associated with educational level, Raven's Standard Progressive Matrices (SPM) score, Wisconsin Card Sorting Test (WCST) category score, and Digit Span Test (DS) in schizophrenia (Table 2). The FER *d'* was correlated to age, SPM score, and WCST category score in nonclinical comparisons (Table 2).

**Table 2:** Correlations between facial emotion recognition performance (FER *d'*), demographic data, clinical characteristics, and neurocognitive measures in schizophrenia and nonclinical comparisons ^a^.

FER *d'*	Correlation coefficient ^a^ (*ρ*)	
	Schizophrenia (N = 74)	Healthy controls (N = 80)
**Age**	0.01	-0.28*
**Educational level, years**	0.25*	0.12
**Clinical characteristics**		
**Age of onset**	0.16	N/A
**Duration of illness**	-0.16	N/A
**Number of hospitalizations**	-0.20	N/A
**Chlorpromazine equivalents (mg)**	-0.035	N/A
**PANSS**		
Positive symptoms	-0.14	N/A
Negative symptoms	-0.05	N/A
General psychopathology	-0.07	N/A
Total score	-0.13	N/A
**CDSS**	-0.09	N/A
**BDI**	N/A	-0.20
**Neurocognitive measures**		
SPM	0.53**	0.26*
CPT *d’*	0.15	0.14
WCST category	0.37**	0.35**
Digit span	0.32**	0.17

^a^ Nonparametric correlation by Spearman *ρ*

PANSS = Positive and Negative Syndrome Scale; CDSS = Calgary Depression Scale for Schizophrenia; BDI = Beck Depression Inventory; SPM = Raven’s Standard Progressive Matrices; CPT = Continuous Performance Test; WCST = Wisconsin Card Sorting Test

*p < 0.05,

**p < 0.01

### Correlational analyses of social function

The social function measures (SFS domain scores) were extensively associated with FER *d'* (*r* = 0.24–0.36), SPM score (*r* = 0.30–0.43), Continuous Performance Test (CPT) sensitivity index (*r* = 0.24–0.36), WCST category score (*r* = 0.27–0.62), DS (*r* = 0.30–0.37), Positive and Negative Syndrome Scale (PANSS) -Positive score (*r* = 0.25), PANSS-Negative score (*r* = 0.24–0.29), and PANSS-General score (*r* = 0.24–0.25) in patients with schizophrenia (Table 3).

**Table 3:** Correlations between social function scores, clinical symptoms, neurocognitive measures, and facial emotion recognition performance in schizophrenia ^a^.

	FER *d’*	SPM	CPT *d’*	WCST category	Digit span	PANSS-positive	PANSS-negative	PANSS-general
**SFS_SE/W**	0.02	0.24*	0.21	0.10	0.05	-0.25*	-0.18	-0.07
**SFS_IPC**	0.24*	0.32**	0.07	0.27*	0.37**	0.08	-0.04	0.25*
**SFS_I/C**	0.36**	0.32**	0.01	0.62**	0.04	-0.07	-0.14	0.24*
**SFS_I/P**	0.28*	0.43**	0.36**	0.27*	0.09	0.08	0.29*	0.25*
**SFS_R**	0.22	0.39**	0.24*	0.11	0.30**	0.09	0.09	-0.16
**SFS_PA**	-0.17	0.09	0.23	-0.22	0.08	0.17	0.24*	0.09
**SFS_E/O**	0.25*	0.30**	0.01	0.08	0.22	-0.04	0.09	0.06
**SFS total**	0.27*	0.39**	0.30*	0.22	0.23	-0.09	-0.08	-0.12

^a^ Nonparametric correlation by Spearman *ρ*

SFS = Social functioning scale; SFS_SE/W = Social engagement/withdrawal; SFS_IPC = Interpersonal communication; SFS_I/C = Independence/competence; SFS_I/P = Independence/performance; SFS_R = Recreation; SFS_PA = Prosocial act; SFS_E/O = Employment/Occupation

PANSS = Positive and Negative Syndrome Scale; FER = Facial emotion recognition; SPM = Raven’s Standard Progressive Matrices; CPT = Continuous Performance Test; WCST = Wisconsin Card Sorting Test

*p < 0.05,

**p < 0.01

### Regression model of global social function

Multivariate regression analyses revealed that years of education, sustained attention (CPT *d’*), age, working memory (DS) and facial emotion recognition performance (EFR *d’*) were significantly associated with global social functioning (SFS total score) in schizophrenia (Table 4). The model was significant with an adjusted *R*^*2*^ of 0.44 (F = 12.073, *p* < 0.001).

**Table 4:** Model of global social functioning (SFS total score) by multiple regression analyses in schizophrenia ^a^.

Predictors of global social functioning ^b^	Standardized regression coefficient (*β*)	*p*
**Education (years)**	0.473	0.000
**CPT *d’***	0.461	0.000
**Age (years)**	-0.295	0.002
**Digit span**	0.333	0.001
**FER *d'***	0.212	0.038

^a^ Model *R*^*2*^ (adjusted) = 0.438, F = 12.073, *p* < 0.001

^b^ Sequence of variables entered into the model was ordered from top to bottom

CPT = Continuous Performance Test; FER = Facial emotion recognition

## Discussion

In the current study, we explored the relationships among social function, clinical symptoms, basic neurocognition, and affect recognition in schizophrenia. Given that functional outcome has clinical significance in schizophrenia, the interest in discovering possible indicators has been mounting in recent years [1].

Nevertheless, the independence/interdependence between emotional perception and neurocognition has long been be debated because of its theoretical importance. Neurophysiological evidence suggests that facial emotion recognition could be an independent neural event via activation over brain regions related to autonomic processing of emotions, such as the limbic structure [31]. However, interconnection between the emotion-processing limbic area and execution-related medial frontal area has also been reported in a study of emotion recognition deficits in schizophrenia [32]. Experimentally, affect recognition deficits in schizophrenia demonstrated abnormalities in early visual encoding of facial features [33], and may also be influenced by impairment in down-stream information processing, including attention and executive function [34–35]. In the present study, we found that facial emotion recognition performance was associated with the SPM score, WCST category score, and DS in schizophrenia. In the nonclinical comparison group, facial emotion recognition performance was also correlated to the SPM score and WCST category score. These results echo experimental data that there may be interdependence between facial emotion recognition and some domains of neurocognition [32, 34–35].

The present study showed that there was no association between facial emotion recognition and either age of onset or duration of illness. The lack of effect of age of onset and duration of illness on the performance of facial emotion recognition may be explained by some evidence that emotion recognition deficits may develop in early stage of schizophrenia [36–37].

Global social function in the current study was significantly correlated to general intelligence, attention, and facial emotion recognition. The findings replicate the results from numerous previous studies in Western countries and Chinese populations [3–5, 8, 38]. Furthermore, we found that the various subdimensions of social functioning were extensively associated with general intelligence, facial emotion recognition, attention, executive function, working memory, positive symptoms, negative symptoms, and general symptoms, with different patterns. For example, we observed that general intelligence was extensively correlated to various domains of social function except for prosocial activities. In contrary, prosocial activities were associated with negative symptoms only. It is worth noting that facial emotion recognition was also correlated to many domains of social function, such as interpersonal communication, independence/competence, independence/performance, and employment/occupation. This finding is in line with existing evidence that deficits in emotion recognition have significant impact on social functioning and outcome in patients with schizophrenia via interpersonal interaction, social skill, quality of life, occupational ability, and independence [1, 38–41].

In this study, we aimed to determine the extent of the contribution of possible factors to social functional outcome in schizophrenia. Considering the complex relationships among clinical symptoms, neurocognition, affect recognition, and social function, it is necessary to take all these factors into consideration simultaneously when studying their functional significance. Using multivariate regression analyses to explore the contribution of these possible factors, we found that global social functioning was best predicted by more years of education, better sustained attention, younger age, better working memory, and better facial emotion recognition performance. The model was able to predict 43.8% of the variance concerning global social functioning in schizophrenia (F = 12.073, *p* < 0.001). Neurocognitive ability significantly contributed to the variances of social functional outcome in addition to emotion recognition performance and basic patient data. Our results are consistent with previous findings, which highlighted the significance of neurocognition in sustaining the abilities required for social functioning [42–43], especially working memory and attention [3, 12, 38]. Unlike another study which found negative symptoms play a role in functional outcome in stable community outpatients with schizophrenia [38], we didn’t find a significant role of clinical symptoms in our subjects, who were a mixed group of outpatients and inpatients.

The task of emotion recognition in the present study mainly measured the early stage of facial affect differentiating and labeling, which may be indirectly related to global social functioning in the real world. However, emotion recognition in this study demonstrated a crucial role, in addition to basic subject data and nonsocial neurocognitive ability, in predicting global social function in schizophrenia. This finding is in line with other studies [7]. Even though some researchers argued that social function impairment in patients with schizophrenia is owing to their failure to realize the meaning of perceived emotional cues rather than disabilities in affect recognition [9], our results support the postulates that difficulties in an earlier phase of emotion perception may have influence on the down-stream process of judging emotional implications [10], thus leading to dysfunction in social problem solving and community functioning.

### Limitations and strengths

The current study has several limitations and a generalization of our results must be viewed with caution. First, due to the cross-sectional design of this study, causal interpretations can only be deduced. Second, the clinical characteristics of our patients were heterogeneous with various durations of illness and mixed outpatient/inpatient status, although all of them were clinically stable under treatment at the time of the study. Also, the educational level in the nonclinical control group was higher than that in the schizophrenia group. Although we selected neurocognitive tasks which are hypothetically correlated to social functioning, data from some other neurocognitive domains such as processing speed, verbal learning/memory, and visual learning/memory are still lacking. Finally, the use of multiple neuropsychological measurements in current study could increase type I error in statistics.

In spite of these considerations, this study has many implications for research and practice. Our study is one of the few to investigate the functional significance of clinical symptoms, neurocognition, and affect recognition simultaneously in patients with schizophrenia.

## Conclusions

The results from the present study indicate that the various subdomains of social functioning are extensively related to general intelligence, basic neurocognition, facial emotion recognition, and clinical symptoms, with different association patterns. Moreover, affect recognition in this study demonstrated a crucial role, combined with basic demographic data and nonsocial neurocognition, in predicting global social function in schizophrenia. Aggressive treatment toward deficits in neurocognition and affect perception, as well as clinical symptoms may be able to ameliorate the functional outcome of patients with schizophrenia. Obviously, further research must be done with a longitudinal study design, more comprehensive measures of possible factors concerning social functioning, and a larger sample size which can afford more detailed subgroup analysis to illuminate the mechanism of social function impairment in schizophrenia. The development and application of more effective pharmacological/non-pharmacological interventions including prevention, treatment, and rehabilitation are required to ameliorate symptoms and improve patient outcomes.

## Supporting Information

S1 Fig Recognition of facial expressions of basic emotions by the control, paranoid schizophrenia, and non-paranoid schizophrenia groups. d′ = Z(hit rate)−Z(false alarm rate), where function Z(p), p∈ [0,1]; *p<0.05, **p<0.01.
(TIF)

S1 Table Correlations between variables. PANSS = Positive and Negative Syndrome Scale; SPM = Raven’s Standard Progressive Matrices; CPT = Continuous Performance Test; WCST = Wisconsin Card Sorting Test; FER *=* facial emotion recognition; *p < 0.05, **p < 0.01.
(PDF)
