# Supplementary figures and images for: The Functional Significance of Affect Recognition, Neurocognition, and Clinical Symptoms in Schizophrenia
Source: PLoS One. 2017 Jan 18;12(1):e0170114. doi: 10.1371/journal.pone.0170114 (PMC5242509; doi:10.1371/journal.pone.0170114)

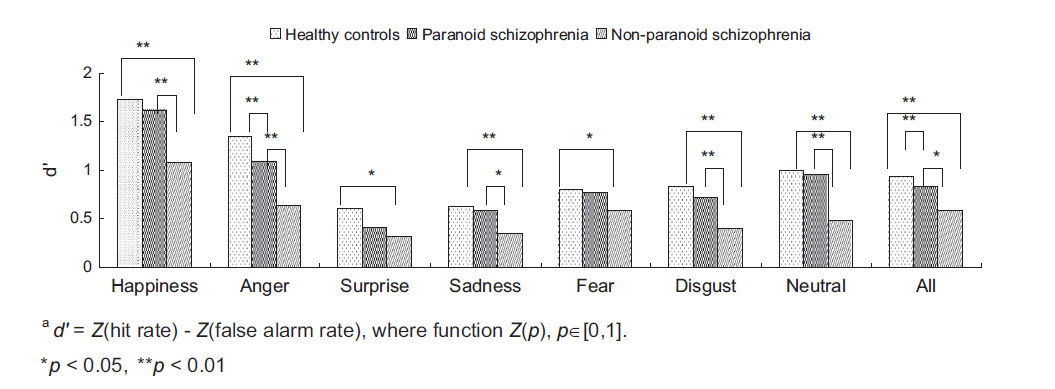

Supplement: S1 Fig — d′ = Z(hit rate)−Z(false alarm rate), where function Z(p), p∈ [0,1]; *p<0.05, **p<0.01. (TIF) [file pone.0170114.s001.tif]
