# Supplementary material for: The Functional Significance of Affect Recognition, Neurocognition, and Clinical Symptoms in Schizophrenia
Source: PLoS One. 2017 Jan 18;12(1):e0170114. doi: 10.1371/journal.pone.0170114 (PMC5242509; doi:10.1371/journal.pone.0170114)
Supplement: S1 Table — PANSS = Positive and Negative Syndrome Scale; SPM = Raven’s Standard Progressive Matrices; CPT = Continuous Performance Test; WCST = Wisconsin Card Sorting Test; FER = facial emotion recognition; *p < 0.05, **p < 0.01. (PDF) [file pone.0170114.s002.pdf]

		SFS_total	Age	Edu_yr	FER_d’	CPT_d’	WCST_category	DigSp	SPM	PANS_sum
Pearson Correlation	SFS_total	1.000	-.245	.410	.120	-.338	.184	.244	.378	-.130
	Age	-.245	1.000	.060	-.137	.062	-.297	.065	-.076	-.069
	Edu_yr	.410	.060	1.000	.258	.103	.528	.169	.272	-.167
	FER_d’	.120	-.137	.258	1.000	-.148	.324	.302	.429	-.196
	CPT_d’	-.338	.062	.103	-.148	1.000	.095	.186	-.055	-.230
	WCST_category	.184	-.297	.528	.324	.095	1.000	.116	.288	-.320
	DigSp	.244	.065	.169	.302	.186	.116	1.000	.487	-.582
	SPM	.378	-.076	.272	.429	-.055	.288	.487	1.000	-.477
	PANS_sum	-.130	-.069	-.167	-.196	-.230	-.320	-.582	-.477	1.000
Sig. (1-tailed)	SFS_total	.	.019	.000	.157	.002	.061	.019	.001	.139
	Age	.019	.	.309	.126	.303	.006	.294	.263	.283
	Edu_yr	.000	.309	.	.014	.194	.000	.078	.010	.081
	FER_d’	.157	.126	.014	.	.107	.003	.005	.000	.049
	CPT_d’	.002	.303	.194	.107	.	.214	.059	.322	.026
	WCST_category	.061	.006	.000	.003	.214	.	.165	.007	.003
	DigSp	.019	.294	.078	.005	.059	.165	.	.000	.000
	SPM	.001	.263	.010	.000	.322	.007	.000	.	.000
	PANS_sum	.139	.283	.081	.049	.026	.003	.000	.000	.
